# Facing Your Fears in Adolescence: Cognitive-Behavioral Therapy for High-Functioning Autism Spectrum Disorders and Anxiety

**DOI:** 10.1155/2012/423905

**Published:** 2012-10-03

**Authors:** Judy Reaven, Audrey Blakeley-Smith, Eileen Leuthe, Eric Moody, Susan Hepburn

**Affiliations:** Anschutz Medical Campus, School of Medicine, University of Colorado, Aurora, CO 80045, USA

## Abstract

Adolescents with high-functioning autism spectrum disorders (ASDs) are at high risk for developing psychiatric symptoms, with anxiety disorders among the most commonly cooccurring. Cognitive behavior therapies (CBTs) are considered the best practice for treating anxiety in the general population. Modified CBT approaches for youth with high-functioning ASD and anxiety have resulted in significant reductions in anxiety following intervention. The purpose of the present study was to develop an intervention for treating anxiety in adolescents with ASD based on a CBT program designed for school-aged children. The Facing Your Fears-Adolescent Version (FYF-A) program was developed; feasibility and acceptability data were obtained, along with initial efficacy of the intervention. Twenty-four adolescents, aged 13–18, completed the FYF-A intervention. Results indicated significant reductions in anxiety severity and interference posttreatment, with low rates of anxiety maintained at 3-month follow-up. In addition, nearly 46% of teen participants met criteria for a positive treatment response on primary diagnosis following the intervention. Initial findings from the current study are encouraging and suggest that modified group CBT for adolescents with high-functioning ASD may be effective in reducing anxiety symptoms. Limitations include small sample size and lack of control group. Future directions are discussed.

## 1. Introduction

Anxiety is one of the most common mental health conditions affecting children and adolescents [1–3]. Children with high-functioning autism spectrum disorders (ASDs) are at high risk for developing cooccurring mental health conditions, and the risk appears to be higher than for children with a history of typical development [4–6]. For example, some studies suggest that while anxiety disorders are estimated to occur in 2.2–27% of the general pediatric population, rates of occurrence of anxiety disorders for youth with ASD are reported to be as much as two times higher [6]. 

Anxiety can be a debilitating disorder for individuals with ASD, potentially impacting individuals across all environmental contexts. Childhood anxiety also has a marked impact on family functioning, to an even greater extent than other social environments [7]. Additionally, excessive worry and distress regarding social situations may compound the core deficits of ASD and prevent the formation of meaningful social relationships, leading to isolation in navigating social environments [8]. School performance may also be affected, as students with anxiety are at risk for academic underachievement, which in turn impacts grades, overall school performance, and participation in after-school activities [9–11]. Others have suggested that because anxiety may in part exacerbate the atypical social behavior of students with ASD, specific interventions may be necessary to fully include students with ASD in regular classroom programming [12]. Thus, over the long term, the presence of anxiety symptoms places individuals at risk for the development of other psychiatric symptoms, limited social supports, difficulties in school, and underemployment [13].

As cognitive behavior therapy (CBT) has been increasingly identified as the gold standard approach for treating mental health symptoms in typically developing youth, researchers have begun applying CBT approaches to other populations, including children with ASD. The results of individual case studies [14, 15], small group studies [16–18], and randomized clinical trials [19–23] have demonstrated reductions in anxiety symptoms after the implementation of modified CBT techniques. Adaptations of CBT have been implemented both individually [23] as well as in groups [20] for youth with ASD with good results. Of the 10 treatment studies cited above, the majority included children age 13 and younger [14, 15, 19, 21, 23]. Two studies included adolescents [17, 18], and three studies included both children and teens [16, 20, 22]. 

In the two randomized trials that included both children and adolescents, the exact number of adolescents who participated in each study is unclear; however, the adolescent participants in both studies were in their early to middle teenaged years (e.g., ages 13–16) [20, 22]. The mean age of the participants in each treatment study was similar (e.g., in the Reaven et al. study, the mean age for the CBT group = 10.48; mean age for treatment as usual group = 10.42 [20]; in the Sung et al. study, mean age for the CBT group = 11.33; Mean age for comparison group = 11.09 [22]). The content of the two treatment programs appears to be fairly similar, as core CBT concepts are included in both programs. Apparent differences include the relative emphasis on graded exposure, use of technology and parent participation in the Reaven et al. program, while the Sung et al. program was specifically designed and modified for an Asian population. 

Thus, of the few treatment studies that have targeted anxiety in youth with ASD, the majority have focused primarily on school-aged children. The limited attention to adolescents is notable, since teens with ASD and anxiety are not only susceptible to the development of anxious symptoms, but the presence of these symptoms may be especially impairing [24]. That is, while adolescence can be a challenging developmental period even for typically developing youth, teens with ASD are navigating a complex social milieu with far fewer psychological resources. Developing friendships and budding romantic relationships, exploring new vocational opportunities, attending colleges and universities, and nearing independent living reflect just some of the tasks of normal adolescence [25–27]. Teens with ASD not only experience similar developmental tasks and challenges, but they are also faced with increased self-awareness of social differences, and with increasingly complex social relationships in the presence of compromised social ability [18, 28]. Teens with high-functioning ASD may be particularly vulnerable to a “perfect storm”—high intellect and a vulnerability to psychiatric symptoms, coupled with few psychological resources and limited access to state/federal supports [29]. 

Treatment programs designed specifically for adolescents with ASD and anxiety need to be further developed, given that simple upward extensions of existing CBT programs for youth with ASD, or even CBT programs designed for typically developing teens, may not be sufficient [27]. The presentation of anxiety symptoms in adolescents may be complex, severe, and chronic, and unique developmental characteristics of adolescents are essential to consider when designing treatment programs for teens [30]. As a result, common adaptations to treatment programs for adolescents have included the use of visually appealing and age appropriate materials, increased interactive activities, and the use of multimedia materials and presentations. The level of parent involvement has varied based on the teen's competency, autonomy, and quality of the parent/teen relationship [30]. 

Another critical issue to consider when working with adolescents is that anxiety symptoms can change over time. For example, while specific phobias may be more common for younger children, symptoms of social phobia may be more apparent in teenagers [3, 31]. In youth with ASD, social anxiety occurs at higher rates compared with typically developing youth [32, 33]. Although the relationship between social deficits and anxiety for youth with ASD is complex, potentially effective intervention programs likely need to emphasize the development of fundamental social skills paired with exposure hierarchies [8, 34], signaling the need for further adaptations to intervention programs for teens with ASD. 

In addition, the role of parents in the treatment of adolescent anxiety also needs to be determined, as the frequency and quality of parent involvement should be tailored to meet the developmental needs of teen participants. In the general pediatric literature, the impact of parental participation in treatments for childhood anxiety has yielded mixed results, with the greatest benefits noted for younger children [35]. In the treatment studies conducted thus far for school-aged children with high-functioning ASD and anxiety, clinical researchers consistently highlight the importance of parent participation. Parents of youth with ASD may have a larger and more constant role than they would ordinarily have for their typically developing offspring given the severe and chronic nature of the core deficits of ASD. Parents serve as advocates, coaches, cheerleaders, friends, and teachers, frequently supporting the generalization of new skills from one setting to another for their children with ASD [27]. Other researchers have suggested that without family support, the cognitive characteristics of youth with autism-related symptoms make it difficult for these youth to access individual CBT interventions [36]. However, the extent to which parent participation enhances the treatment benefits for adolescents, particularly adolescents with ASD, is unknown. In fact, parent participation has not always been included as part of the treatment package for teens with ASD and anxiety in previous studies [22]. The balance between the teens' need for independence and the reality of their ongoing dependence on their parents, especially when compared to their typically developing counterparts, needs to be delineated. Challenges in parent-child agreement of anxiety symptoms [37, 38], combined with difficulty generalizing skills beyond the treatment setting, provide further support for the continued presence of parents in teen treatment. 

Finally, teens with ASD may be resistant to participation in conventional psychosocial interventions. Therefore, innovation in intervention structure and content may be critical in peaking teens' interests. Given the intense interest that adolescents with ASD often have in technology, capitalizing on this interest through self-monitoring methods may be a useful addition to treatment [39]. Increased motivation and interest may result in increased responsiveness to the intervention. Several recent papers have supported the integration of technology with traditional models of psychotherapy [40, 41]. 

The purpose of the present study was to modify our treatment program for children with high-functioning ASD and anxiety: Facing Your Fears: Group Therapy for Managing Anxiety in Children with High-functioning ASD (FYF; [42]). Recently published results from a randomized controlled trial indicated that children who received the FYF intervention demonstrated significant reductions in anxiety severity, had fewer anxiety diagnoses postintervention, and were more likely to experience clinically meaningful improvement in symptoms postintervention compared to participants assigned to the Treatment As Usual (TAU) condition [20]. 

Given the initial success of FYF for children ages 8–14, expanding the intervention, while simultaneously considering the unique developmental needs of teens with ASD, was a logical next step. An initial pilot study of teens with high-functioning ASD and anxiety yielded promising results [17], but due to the small sample size (*n* = 4), further study was required. Thus, the overall purpose of the present study was to build on our previous work by further developing and manualizing a CBT group treatment specifically designed for adolescents with ASD and anxiety. There were several unique aspects to our adolescent treatment program. We were particularly interested in developing a parent component to the intervention package, since other treatment programs have not consistently included parent involvement [22]. Furthermore, unlike other anxiety treatment programs for teens with ASD, we also integrated the use of technology for the delivery of core CBT components and monitoring of anxiety symptoms via a handheld device (e.g., Palm Z22 PDA; Apple iPod Touch). An additional purpose of the present study was to assess the feasibility and acceptability of such an intervention, and to examine the initial efficacy of the intervention with a small group of teens with ASD and anxiety. It was hypothesized that adolescents participating in the FYF- Adolescent Version (FYF-A) would display significant reductions in anxiety symptoms after participating in the intervention. 

## 2. Method

### 2.1. Participants

Participants were recruited through the Colorado Multiple Institution Review Board (COMIRB) approved study announcements, which were mailed to local parent groups, high schools, and clinics. Informed consent and assent were obtained for all participants prior to collecting any data. Thirty-five adolescents and their parents enrolled in the treatment study in 2008–2010. See Table 1 for participant characteristics.

Of the 35 families that contacted the research clinic, four youth did not meet the inclusion criteria. Thirty-one teens and families were invited to join the treatment study. Five families dropped out of the study between qualification and intervention (one moved, one underwent a medical procedure, one teen declined participation, and two experienced scheduling difficulties). Twenty-six entered treatment, but two adolescents dropped out prior to session two (one was hospitalized for psychiatric treatment out of state, one required individual treatment for a traumatic event that occurred just after the qualification period and prior to the beginning of group). Twenty-four families completed the 14 session intervention program. All families who did not participate in the intervention were given appropriate mental health referrals. 

Inclusion criteria for teen participants were (1) *chronological age from 13–18 years;* (2) *a confirmed diagnosis of an ASD, *as determined by one of two expert clinical psychologists (SH, ABS) based upon review of a recent (within one year or newly administered) Autism Diagnostic Observation Schedule (ADOS; [43]) and the Social Communication Questionnaire (SCQ; [44]); (3) *speaking in full, complex sentences, *defined as the ability to complete either Module III or IV of the ADOS; (4) *estimated verbal IQ of 70 or higher, *as determined through standardized cognitive testing using the Wechsler Abbreviated Scales of Intelligence (WASI; [45]) or an equivalent measure of intelligence administered within two years prior to recruitment; (5) *clinically significant symptoms of anxiety*, defined as a score above the clinical significance cutoff on separation (SEP) social (SOC) and/or generalized anxiety (GAD) subscales of the Screen for Child Anxiety and Related Emotional Disorders (SCARED)—parent or child versions [46]. 

Participants were excluded if (1) the teen's primary psychiatric symptoms included marked depression or other mood symptoms, psychosis, or severe aggressive behavior, as determined through results of the Anxiety Disorders Interview Schedule for DSM-IV: Parent and Child Versions (ADIS-P and ADIS-C; [47]). In other words, the diagnosis with the highest clinician-assigned Clinical Severity Ratings (CSRs) was considered “primary.” In the event of a tie, parents were asked to indicate which set of symptoms was most problematic for their child. The presence of significant symptoms in these areas may have indicated that a more intensive or different treatment approach would be required; (2) if the teen did not demonstrate “group readiness” in the first three sessions of FYF-A. For example, the inability to separate from a parent, aggressive outbursts, or chronic resistance to treatment are potential indicators of a lack of group readiness; (3) if one parent could not commit to attending at least 11 of 14 sessions. 

Adolescents currently taking medications were eligible for the study, but families were asked to consult with the prescribing physician prior to enrolling in the study to stabilize medication dosages at least two weeks prior to the initial assessment. Families were asked to keep the type and dosage of medications consistent throughout the duration of their participation (approximately 8 months; baseline through treatment and 3-month follow-up). Medications and dosage information were documented on a weekly basis. 

### 2.2. Procedure

Study procedures were completed in compliance with COMIRB. Families initiated contact with the research clinic and obtained information regarding the goals and requirements of the study. If the study was of interest to the parent, a brief telephone screen for eligibility was conducted by a research assistant. If the teen was a potentially eligible participant, the family was invited to the research clinic and provided with informed consent/assent prior to collecting any data. Teen assent was necessary for study inclusion. Once consented, the family participated in one or two assessment sessions to complete the qualification battery, which included assessment to confirm a diagnosis of an ASD, standardized cognitive testing (if not available either through previous research visits or school testing), the SCARED [46] (completed separately with parents and teens) to screen for significant anxiety symptoms, and additional measures not reported here (e.g., the Revised Children's Manifest Anxiety Scale [48, 49]; the Multi-dimensional Anxiety Scale for Children [50, 51]; the Developmental Behavioral Checklist [52]; and the Children's Automatic Thoughts Scale [53]). These measures were included in the original assessment battery to further understand the assessment and measurement of anxiety in adolescents with ASD, and because of the scope of the topic, will be presented in a second manuscript that is currently underway. A family was determined to be eligible on the qualification battery if parent or teen report indicated scores above the clinical significance cutoff on separation (SEP), social (SOC), and/or generalized anxiety (GAD) subscales of the SCARED. The parent and teen were then invited for an additional session to complete the ADIS (parent and teen report; [47]). Families were compensated for their participation in the assessment. All consented youth met criteria for risk of anxiety symptoms. Parent and teen acceptability of the intervention was obtained after completing the intervention. Within six weeks of the last session, the anxiety measures (i.e., SCARED, ADIS-P) were readministered. 

Six groups, comprised of 3–5 families (mode = 4), were conducted during the two-year study period. The study was initially considered to be a development project, with the goal of creating an adolescent version of FYF. In addition, a related goal was to determine the feasibility and acceptability of the expanded FYF program for adolescents. It was anticipated that multiple iterations to the intervention would occur after each cohort of teens and parents completed treatment. While facilitator debriefing occurred postintervention for each of the six treatment groups, substantial changes to the treatment program did not occur as a result of the debriefing. Rather, only relatively minor changes were made to the intervention (e.g., order of the components presented). Although efforts were made to ensure that all groups received the core CBT components, we cannot make statements regarding the absolute session-by-session intervention fidelity.

**Attendance**

Of the 24 families who completed treatment, 7 families attended 100% of group treatment sessions, 13 attended 92.8% of sessions, 3 attended 85.7% of sessions, and 1 attended 78.6% of sessions. Therefore, only one family attended less than 80% of sessions and required more than one “make-up” session (e.g., coming 20–30 minutes early after missing a session to “catch up” on missed material with a therapist).

**Facing Your Fears-Adolescent Version (FYF-A)**

Each 90 minute group session included large-group activities (teens and parents together), small-group activities (teens alone; parents alone), and dyadic work (parent/teen pairs). There were a total of 14 sessions, plus one booster session held 4–6 weeks after the 14th session. Two clinical psychologists led each group (JR and ABS), supported by two cotherapists (trainees in clinical psychology). Seven total facilitators led the six groups over the study period. Facilitators new to the project were instructed to read the original FYF program, as well as several relevant research articles, and to participate in biweekly supervision sessions with the coauthors.

The original FYF intervention was written and developed specifically for children (ages 8–14) with ASD. The FYF program was comprised of core cognitive behavior therapy (CBT) components drawn from prior empirically supported programs (e.g., *Coping Cat*; [54]), while making appropriate adaptations for youth with high-functioning ASD. Key components such as graded exposure, somatic management, strategies for emotion regulation, and use of cognitive self-control [55, 56] were included in the FYF intervention. 

Similar to the original FYF program, the 14 multifamily group sessions of the FYF-A included (1) an introduction to anxiety symptoms and common CBT strategies, (2) a focus on the implementation and generalization of specific strategies to treat anxiety (i.e., expanding calming activities, recognizing automatic negative thoughts, developing coping statements, and engaging in graded exposure tasks—or facing fears a little at a time). Modifications in the delivery of therapeutic content were recommended because youth with ASD typically have difficulties with self-regulation, exhibit rigid thought processes, have poor social understanding, and demonstrate limited capacity to generalize [57, 58]. Thus, in efforts to enhance the accessibility of the intervention, the original FYF program included careful pacing of each session, visual structure and predictability of routine, written worksheets with multiple choice lists and examples of core concepts, hands-on activities, a focus on strengths and special interests, multiple opportunities for repetition and practice, video modeling activities, and a detailed parent curriculum [42]. 

The FYF-A expanded upon the modifications that were developed when working with younger children in the original program, but additional changes occurred to accommodate the needs of teenagers with ASD: (1) a specific social skills module was developed (see below) to address deficits in social skills functioning; (2) parent-teen dyadic work was included to identify primary anxiety diagnoses and related goals; (3) technology was incorporated in the form of a personalized digital assistant (see below), (4) number of in-session exposure practices was increased and occurred almost exclusively within the adolescent group and without direct parent involvement in-session; (4) the parent curriculum was modified to focus on the unique developmental challenges of adolescence, in addition to the provision of psychoeducation of anxiety and overview of CBT techniques and strategies.

**Social Skills Module**

A specific social skills module was developed to provide opportunities for the teens to address common areas of social challenge (e.g., engaging in conversation, joining a group, or advocating for self), which were thought to underlie the symptoms of social anxiety and were common areas of deficit in teen participants. By directly targeting social skills in addition to implementing core CBT, the FYF-A program used a two-pronged approach to address social anxiety symptoms (e.g., skill building combined with graded exposure). The social skills module consisted of a three session block presented in the first few weeks of the intervention. Teens were initially presented with a list of 12 common social difficulties based on preassessment ADIS data (e.g., interacting in a group, handling teasing, asking a teacher for help, or talking to unfamiliar peers or adults) and were asked to select the top 6 situations they wanted to target in group. Group facilitators selected the most highly ranked situations based on teen responses. Brief role-playing scenarios were created for each of the targeted situations, and the teens took turns taking on different roles. Each of the “players” was given written suggestions for how to enact the role-play and were assigned coaches (other peers or group facilitators), to offer suggestions or helpful hints when they became “stuck.” All role-plays were videotaped, and the teens critiqued the segments as a group. Where applicable, teens were given the opportunity to practice the skills in more naturalistic environments onsite. Parents were also involved in teen social skills development; they identified priority skills for their teen (e.g., improve nonverbal communication, improve conversational skills, etc.) and learned specific strategies to enhance their teen's skill development and generalization.

**Parent-Teen Dyadic Work: Identifying Primary Diagnostic Status and Related Goals**

While less time was spent in parent/teen dyads in order to promote cohesion and support among the separate teen and parent groups, the time that was spent together was focused on achieving shared understanding and goals. The teen participants were a psychiatrically complex group of individuals. The majority of the adolescents carried more than one diagnosis, including multiple anxiety diagnoses (see below). In order to determine the priority symptoms for interventions (among the myriad of presenting symptoms), at the beginning of the intervention, parents and teens were presented with a list of symptoms from both the ADIS-P and ADIS-C that reflected their primary anxiety diagnosis. Primary diagnosis was defined as the anxiety diagnosis receiving the highest CSR from the ADIS. This activity provided parents and teens the opportunity to identify target anxiety symptoms and to establish exposure hierarchies directly related to the teen's primary diagnosis.

**Use of Technology**

A hand-held PDA was introduced to the teens early in the program (Session 4) to complement the core components of the intervention. The purpose of the PDA was to assist the teens in regularly monitoring their anxiety symptoms, to remind the participants to engage in relaxing or calming activities on a daily basis, to guide participants through a series of coping strategies when stressed, and to document exposure practice. A Palm Z22 PDA was used for the first half of the study (*n* = 12), and an iPod touch was used for the second half of the study (*n* = 12). (The hand-held devices served similar functions, but an iPod touch was added half-way through the study because of changes made by Symtrend, the company that provided software for the PDAs.)

**Parent Component**

The parent component of FYF-A included the following: (1) psychoeducation; (2) parent coaching; (3) a focus on the interaction between parental anxiety, parenting style, and the maintenance of anxiety symptoms; (4) discussion of the social/communicative challenges inherent in ASD and how these challenges may contribute to a protective parenting style [27, 35]); (5) enhancing teen social skills development. Parents were also encouraged to discuss parenting challenges unique to raising teenagers with ASD. Supportive connections between family members were forged, as the group format promoted parent-to-parent input and discussion. Opportunities for positive interaction between parents and teens were created by emphasizing teen strengths and increasing parents' understanding that frequent displays of teen resistance could be related to long histories of social rejection, rather than lack of motivation or interest [27].

### 2.3. Measures

There were three sets of measures: (1) qualifying battery; (2) outcome battery; (3) process measures (e.g., assessment of acceptability, feasibility, and medication status). 

#### 2.3.1. Qualifying Battery (Included Measures Necessary to Determine the Teen's Eligibility for the Study)

**Diagnosis of ASD**

Diagnostic status was determined by expert clinical review of the Autism Diagnostic Observation Schedule (ADOS; [43]) and the Social Communication Questionnaire—Lifetime Version (SCQ; [44]), as documented using a symptom checklist based on the DSM-IV TR [59]. In the event that a participant did not exceed cutoffs on the ADOS (*n* = 3) and/or the SCQ (*n* = 10), two expert clinicians independently reviewed these cases to determine whether the participant met diagnostic criteria for an ASD. Only one participant did not exceed cutoff on both the ADOS and SCQ; however, it was determined via clinician review that he did meet diagnostic criteria for an ASD.

The ADOS [43] is a semistructured, play-based direct child assessment of social and communicative behaviors indicative of autism. Currently, the use of the ADOS is recommended as best clinical practice in diagnosing autism spectrum disorders, in combination with parent interview [60]. All laboratory personnel met research reliable administration criteria on the ADOS prior to the onset of the study and achieved 85% reliability on the full range of scores. Assessments were videotaped and reliability was assessed for 20% of the 35 ADOS administrations. 

The SCQ [44] is a parent report measure tapping historical and current symptoms of autism. This 40-item, yes/no checklist was empirically derived from the Autism Diagnostic Interview-Revised (ADI-R; 61]) and has been demonstrated to be an effective parent report tool of autism symptoms after the age of 4. This tool requires 20 minutes to complete. The SCQ has good specificity (.80) and sensitivity (.96) [40].

**Intellectual Functioning**

The Wechsler Abbreviated Scales of Intelligence (WASI; [45]) is an IQ screening measure that provides an estimate of the child's verbal and nonverbal potential in a brief period of time. This tool has been shown to provide scores that are reliable with a full battery (i.e., the Wechsler Intelligence Scale for Children IV (WISC-IV; [61])) in samples of children with autism spectrum disorders [61]. Scores from other standardized cognitive tests (such as the WISC-IV;) were also accepted if the adolescent had completed the intellectual assessment within two years by a licensed examiner under standardized conditions.

**Clinical Anxiety Symptoms**

The Screening for Childhood Anxiety and Related Emotional Disorders (SCARED; [46]) is a 41-item inventory of statements that relate to five types of anxiety experienced by children and adolescents, including subdomains of panic, generalized, separation, social, and school anxiety symptoms. There are identical parent and youth versions. A total score, as well as cutoffs for each domain score, is obtained. A total score of 25 or higher indicates risk of anxiety symptoms that interfere with teen functioning.

#### 2.3.2. Outcome Battery (Included Pre- and Postintervention Measures That Assessed the Severity and Interference of Anxiety Symptoms, According to Parent Report and Teen Self-Report)

**The Anxiety Disorders Interview Schedule for DSM-IV**

Parent and Child Versions (ADIS-P and ADIS-C [47]) were administered to both parents and teens in separate clinical interviews (for the purpose of the present study, only parent report will be described). The ADIS is considered to be a best-practice, semi-structured psychiatric interview that assesses the presence of anxiety disorders as well as other psychiatric disorders. The ADIS-P has been used in treatment studies for youth with and without ASD [3, 20]. Clinicians reviewed all data, assigned DSM-IV diagnoses, and determined summary codes of severity and interference, called “Clinical Severity Ratings” (CSRs). CSRs for this study were rated on a 8-point scale, with higher scores indicative of greater impairment (0 = no symptoms, 8 = severe impairments). To achieve reliability on the ADIS, all administrators were required to score above 80% on diagnostic classifications and CSRs for all diagnoses on three videotaped administrations and three live administrations of the ADIS (serving as the interviewer). Two psychologists and two postdoctoral fellows served as administrators of the ADIS. Assessments were videotaped and reliability was obtained on a subsample of 20.8% of the cases.

The Clinical Global Impression Scale-Severity ratings (CGIS-S; adapted from [62]) were compiled pre- and posttreatment, resulting in a single global severity rating for the four anxiety diagnoses. Two clinical psychologists reviewed a deidentified record that included the ADIS-P and the SCARED data. The psychologist provided a rating on a scale of 1–7 of overall symptom severity (1 indicated “not at all ill,” indicated “moderately ill,” and 7 indicated “extremely ill”). Number of diagnoses combined with CSRs for each of the assigned diagnoses determined overall severity (adapted from [62]).

The Clinical Global Impressions Scale-Improvement ratings (CGIS-I; adapted from [63]) were compiled by two clinical psychologists after the posttreatment data were collected in a manner similar to the methods described in previous studies [3, 20, 23]. The psychologists reviewed deidentified records that included the ADIS-P and the SCARED data collected pre- and posttreatment. Ratings were provided on a scale of 1–7 regarding overall impression of improvement during that time period (1 indicated “very much improved,” 4 indicated “no change,” and 7 indicated “very much worse”). CGIS-I scores of 1 or 2 were considered to be positive treatment responders. Of the two psychologists who assigned the improvement ratings, one was a cofacilitator of the treatment groups, and the other was not. The majority of ratings were made by the psychologist who did not cofacilitate the intervention. 

Two clinical psychologists completed the severity and improvement ratings, and interobserver reliability was calculated for 25% of the sample. Intraclass correlation coefficients (ICCs) were calculated for CGIS-S ratings at each time point and for CGIS-I ratings for the improvement in primary diagnosis. ICCs ranged from .80 to 1.0 with a mean agreement of .95 across all ratings. The reliability rater never disagreed more than one point. 

#### 2.3.3. Process Battery

**Acceptability Measures**

Teens and parents completed a brief acceptability measure at the end of the 14-week program to obtain feedback regarding the content of the program and the extent to which the participants found the activities to be helpful. The measure required participants to rate all core activities on a Likert scale from 1–5, reflecting the extent to which they considered each of the activities helpful (1 indicated “not very helpful,” 3 indicated “somewhat helpful,” and 5 indicated “very helpful”).

## 3. Results

**Psychiatric Complexity of Participants**

The 24 participants who completed treatment were a psychiatrically complex group, according to the results of the ADIS-P. The number of psychiatric diagnoses for the teen participants, in addition to ASD, ranged from 2–11 (Mode = 4). The most common diagnoses were Generalized Anxiety Disorder (88%), Specific Phobia (88%), and Social Phobia (88%), followed by Attention Deficit Hyperactivity Disorder (67%) and Obsessive Compulsive Disorder (46%). Thirty-three percent met criteria for Disruptive Behavior Disorders.

**Acceptability of the FYF Intervention**

Adolescents and their parents completed a satisfaction questionnaire post-intervention. Twenty-three teens and 24 parents completed the measure within two weeks of finishing the intervention. Parents rated 15 different activities and teens rated 14 different activities (see Table 2).

Of the 15 parent activities, 14 (93%) were rated as “helpful” or “very helpful.” Of the 14 teen activities, 13 (93%) were rated as “somewhat helpful” or higher.

**Medications and Other Interventions**

Twenty-one of the 24 teen participants did not receive psychiatric intervention services outside the current protocol. Three, however, did receive additional therapy services, but they were not anxiety specific; one teen underwent a brief psychiatric hospitalization during the intervention (Session 3) and added family therapy to his treatment regimen (session 6); one teen added individual support from a school psychologist (session 7); and one teen added family therapy (Session 5).

Fourteen of the 24 participants were on medication over the course of the intervention. Given the psychiatric complexity of this population, it is difficult to determine the specific symptoms that these medications were prescribed to target (e.g., some medications targeted multiple symptoms). However, the most common class of medications was SSRIs (9 of 14 participants). SSRIs were prescribed either alone (3 of 9 participants) or in combination with a stimulant (1 of 9), mood stabilizer (1 of 9), alpha blocker (1 of 9), and antipsychotic (1 of 9). Families were specifically requested not to change medications during the study (baseline through 3-month follow-up); 7 of the 14 families reported no changes. Two families eliminated medication, three families added a new medication, and two families reduced the dosage of one medication and increased the dosage of another.

**Technology**

The first 12 adolescent participants received the Palm Z22 PDA, loaded with Symtrend software and screens specifically created for FYF-A. Once teens received the PDA (Session 4), they were asked to “check in” on a daily basis and record anxiety levels, document exposure practice (introduced Session 7), and respond to daily reminders to engage in calming/relaxing activities. Half-way through the project, use of the Palm Z22 was discontinued in favor of an Apple iPod touch because of changes made through Symtrend. Results indicated that participants (*n* = 12) using the iPod touch checked in significantly more frequently (*M* = 75.00) than participants using the Palm Z22 PDA (*M* = 36.67); *t*(22) = 1.947, *P* = .03). However, there were no significant differences between the two devices with regard to number of documented exposure practices (*M* = 14.58-Palm Z22; *M* = 12.58-iPod touch; *t*(22) = .63, *P* = .73).

Spearman Rank Order correlation was used to assess the relationship between the frequency of exposure practice and improvement on primary diagnosis (see below). Documented exposure practice was positively but weakly correlated with the improvement in primary diagnosis (*r*_*s*_ = .20, *P* = ns). There was no difference in the relationship between these variables when examined for the Palm Z22 and iPod touch separately. However, these results may be underpowered due to the relatively small sample size. 

 Technological difficulties occurred throughout the project. For example, regular and ongoing documentation was hampered by teen misplacement of the devices and occasional locking of the devices, preventing usage of the Palm Z22 or iPod touch. Although the devices were reset during the next group therapy session, ability to document exposure practice and daily check-in was compromised for some participants. No teen lost the device, and only one device was broken during the course of the study (iPod touch was accidently dropped down an elevator shaft); this device was replaced.

### 3.1. Treatment Outcome

Treatment outcome was assessed using the following variables: (1) CGIS-Severity ratings [62]; (2) CGIS-Improvement ratings [63]; and (3) parent report and teen self-report on the SCARED [46].

**Clinical Global Impression Scale-Severity (CGIS-S)**

Parent report on the ADIS and SCARED were used for CGIS-S and CGIS-I ratings, in keeping with other research studies that have deferred to parent report of child symptoms [8, 16, 19, 21, 23] and based on the recommendation of a recent study examining the use of self-report in adolescents with ASD [38].

The CGIS-S score ranging from 1–7 was assigned by two psychologists who reviewed deidentified records using ADIS-P and SCARED data. Severity ratings were not normally distributed, so Wilcoxon-signed rank tests were used to assess changes in severity. There was a significant difference in clinician rating of anxiety severity, based upon parent report (*Z* = 2.53, *P* = .011). In other words, overall severity of the teen's anxiety symptoms significantly decreased postintervention.

**Clinical Global Impression Scale-Improvement (CGIS-I)—Primary Diagnosis**

CGIS-I scores of 1 or 2 reflected a clinically meaningful improvement in anxiety symptoms and severity. The CGIS-I is a global measure of improvement, and it has been used in previous research with youth with ASD [20, 23]. Nearly 46% of teen participants (11 of 24 participants) obtained a CGIS-I score reflecting a positive treatment response, 33% (8 of 24 participants) obtained a CGIS-I score indicating “some” improvement, 21% (5 of 24 participants) obtained a CGIS-I score indicating “no change” in symptoms, and no participants' symptoms worsened post-intervention (Cohen's *d* = .90).

**SCARED—Parent and Adolescent Self-Report**

**Figure 1:**
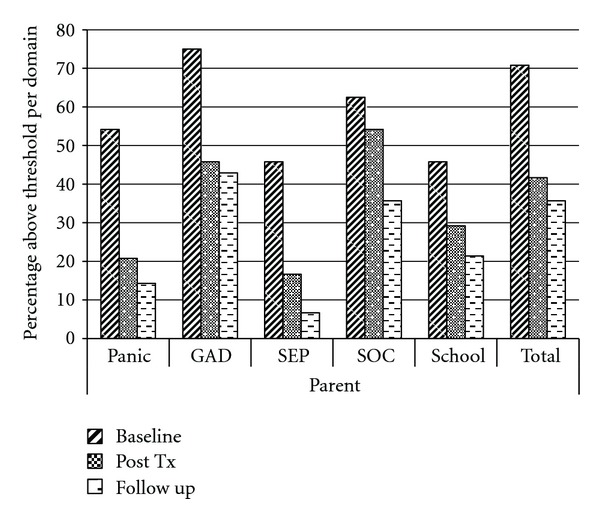
Parent report on the SCARED at baseline, postintervention and 3-month follow-up.

The SCARED was administered to both parents and adolescents at three time points: pretreatment, posttreatment, and 3-month follow-up. All total scores were normally distributed and had excellent internal consistency (Cronbach's alpha ranged from .93 to .95). All paired *t*-tests revealed significant reductions in total anxiety symptoms as reported by parents from pretreatment to posttreatment (*t* = 2.875, *P* = .009) and from pretreatment to follow-up (*t* = 3.821, *P* = .001). Significant reductions in anxiety symptoms were also reported by teens from pretreatment to posttreatment (*t* = 3.896, *P* = .001) and from pretreatment to follow-up (*t* = 3.032, *P* = .008). Visual inspection of the anxiety subdomains on the SCARED indicated that parent-reported scores decreased from pre- to postintervention, with further decreases in symptoms noted for all 5 subdomains at 3-month follow-up (see Figure 1).

**Figure 2:**
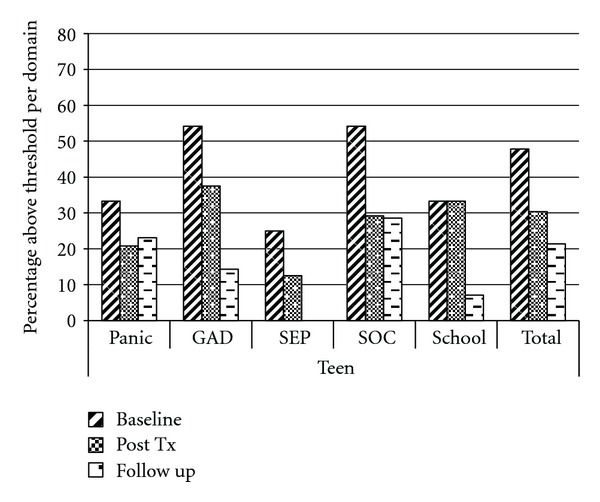
Teen report on the SCARED at baseline, postintervention and 3-month follow-up.

Teen self-report on the subdomains of the SCARED indicated that scores decreased for 3 subdomains from pre- to postintervention, and further decreased at 3-month follow-up (Generalized Anxiety, Separation Anxiety, and Social Anxiety; see Figure 2). In the subdomain of Separation Anxiety, no teens met clinical threshold for anxiety at follow-up. For one of the subdomains (Panic), scores decreased at postintervention, but slightly increased at follow-up, although scores continued to be lower than pretreatment values. Finally, for the subdomain of school anxiety, there was no change between pre- and postintervention, but scores at 3-month follow-up fell very low, such that fewer than 10% of participants continued to meet clinical threshold, compared with 33% at pretreatment. 

## 4. Discussion

The purpose of the current study was to expand on our previous work by developing and manualizing a group treatment for adolescents with high-functioning ASD and anxiety. Ninety-two percent of participants (24 of 26) who entered the 14-week intervention completed the intervention. The two participants who dropped out prior to treatment completion did so by the third treatment session, citing the adolescents' serious psychiatric needs, which required more intensive clinical services than FYF-A could provide. The 24 families who completed treatment displayed excellent attendance, with only 1 family missing more than 80% of total sessions (missed sessions were made up). The results of the satisfaction questionnaires indicate that parents participating in FYF-A found the vast majority of core activities to be helpful or very helpful. Teen satisfaction ratings were slightly lower than for parents; they rated the majority of the core activities as “somewhat helpful” or higher. Taken together, however, these factors indicate that FYF-A may be a feasible and acceptable intervention for teens on the autism spectrum (and their parents) with clinical symptoms of anxiety. 

 Three treatment outcome variables were used to assess the initial efficacy of the FYF-A intervention: (a) change in overall anxiety severity, according to clinician ratings based on parent interview on the ADIS-P; (b) global ratings of improvement of primary diagnosis, according to clinician ratings of improvement based on the ADIS-P; (c) parent/teen report of anxiety symptoms on the SCARED, postintervention and at 3-month follow-up. The results indicated that there were significant reductions in global ratings of anxiety severity and interference following participation in the FYF-A. Nearly 46% of teens met criteria for substantial positive treatment response (Improvement ratings of 1 or 2) for primary diagnosis, while 33% of teens “somewhat improved.” Approximately 21% of teen participants' symptoms remained the same, and no teen participants' symptoms worsened after treatment. Total anxiety symptoms decreased significantly according to parent and teen report on the SCARED. Visual inspection of the subdomain scores for parent and teen report indicated that the vast majority of subdomain scores decreased posttreatment for both parent and teens; further decreases were noted at 3-month follow-up. 

Overall, these results are generally consistent with our own work [20], as well as the work of other clinical researchers [3, 18]. Generalizability of these findings is limited to adolescents who complete the entire 14-week intervention and whose symptoms do not require psychiatric hospitalization. It is notable that other researchers have demonstrated more robust findings with a school-age sample (ages 7–11) following participation in modified CBT delivered individually (e.g., 78.5% of participants met criteria for a positive treatment response; [23]). It may be that the current sample was more psychiatrically complex than in previous studies, as the number of cooccurring psychiatric diagnoses for the teen participants ranged from 2–11 *in addition to* ASD, while the diagnoses of the Wood et al. [23] sample ranged from 2–6 *inclusive* of ASD. In addition, the primary diagnoses that were most commonly identified for the adolescent participants were GAD and social phobia. Targeting these symptoms in adolescents with ASD may require more intensive interventions in order to be most efficacious.

Additionally, adolescents with ASD and cooccurring psychiatric conditions may respond differently to psychosocial interventions relative to their younger counterparts, suggesting that age-related factors may be at play. Family factors are also important to explore, as there may also be real differences between parents of younger children on certain variables (e.g., optimism and expectations of response to interventions; family stress) compared with parents of adolescents. For example, parents of adolescents with ASD typically do not experience a lessening of parental responsibilities and are often handling the myriad challenges of their teens: the development of friendships and romantic relationships, working and living independently, attending college, avoiding victimization, and achieving financial independence [27]. Further, teens with ASD may present with a learning history characterized by multiple failures and rejections [64], suggesting that even the most sensitive and caring parents may experience resistance during interaction with their teens [27]. In fact, interactions between parents and adolescents in the current study were frequently characterized by disagreements on symptom severity, symptom selection, and compliance with check-in and exposure practice. High levels of expressed emotion (e.g., high levels of criticism by one family member towards another; [65]), coupled with an urgency to produce change and solve problems quickly in light of the teen's impending transition to adulthood, may further complicate family interactions. 

 Finally, more individualized and naturalistic approaches toward social skills development [18, 23] may be warranted to increase positive treatment responses among teen participants. For example, in previous research programs [23], clinician researchers created a specific school intervention module to tackle the social skills deficits present in children with ASD. The module focused on friendship skills and included social coaching provided by the therapist, parents, and available school providers, reflecting a coordinated effort between home and school. This approach may be particularly important given the high cooccurrence of social phobia in teens with ASD. 

A novel addition to the current intervention was the use of technology. Because of logistical issues, half the participants received a palm pilot and the other half received an Apple iPod touch. Results indicated that participants who received the iPod touch had significantly more check-ins over the course of the program compared with participants who received the conventional Palm Z22, perhaps reflecting the popular nature of the iPod touch. However, there were no differences between the use of the two hand-held devices with regard to the documentation of exposure practice. In addition, the amount of exposure practice was positively but weakly correlated with the degree of improvement in primary diagnosis (identified by the parent). Lack of a stronger relationship between documented exposure practice and primary diagnosis could be because of the small sample size and resulting lack of statistical power. There may be additional explanations. For example, it may be that adolescent participants documented but did not actually engage in exposure practice, perhaps in an effort to please their parents and/or therapists. Additionally, teens may have had difficulty selecting appropriate exposure activities; that is, they may have selected graded exposure steps that were too easy and/or less related to the actual fears the teens were facing. Finally, as noted above, technological difficulties with the hand-held devices may have impacted data collection, leading to underreporting of the actual number of exposure practices. 

## 5. Limitations and Future Directions

The results were limited by the small sample size and quasi-experimental design. There was no control group: all adolescents and their parents received FYF-A and participants' outcomes were compared to their own baseline. In addition, absolute session-by-session intervention fidelity was not calculated, leaving open the possibility that participants in different groups may have received slightly different intervention components. The extent to which reductions in the severity of anxiety symptoms were related to a bias towards positive changes is unknown. Although charts were deidentified when evaluators assigned CGIS-Severity and Improvement ratings postintervention, it was understood that all the adolescents participated in FYF-A, perhaps biasing results in a favorable direction. Although participants were asked to maintain stable dosages of medications throughout the study, 33% of participants did change medications during the project. Although several of these changes appeared to be unrelated to anxiety symptoms, the extent to which these medication changes truly impacted treatment outcome is unclear. Finally, only the data from the 24 treatment completers was included in the analysis. Two participants dropped out prior to session two of treatment because a different modality of treatment was deemed more appropriate given the severity of their symptoms (e.g., inpatient psychiatric hospitalization, individual psychotherapy for trauma). However, excluding their preassessment data may have artificially inflated the results and should be considered an additional limitation. 

The primary purpose of this study was to develop and manualize FYF-A, a group cognitive behavior therapy program for teens with high-functioning ASD and anxiety. Because FYF-A is an extension of a CBT treatment program for school-aged children with ASD and anxiety (FYF), modifications in the intervention accounting for the cognitive, linguistic, and learning styles of youth with ASD were already in place. However, additional changes to FYF-A were made to take into account developmental differences germane to adolescents. More research is needed in this area to determine the critical elements in intervention that may be unique to adolescents on the autism spectrum. For example, determining the balance between social skills development and core cognitive behavior therapy components, as well as the dosage of the key elements needs to occur. The role of parent involvement in treatment protocols for teenagers with ASD should also be explored, given the ongoing advocacy and caretaking role of many parents. Incorporating skill development within the context of naturalistic environments should also be considered to strengthen treatment protocol. The introduction of PDAs into the intervention program represented a protocol addition that was specifically designed to increase adolescents' motivation and interest. While teen participants did use the PDAs (the iPod touch in particular), more research is needed to explore the multiple functions of the PDAs and how these functions can be linked directly to the core treatment components. 

## 6. Conclusions

 The present study is one of only a few studies to date targeting adolescents with high-functioning autism spectrum disorders and anxiety. The results of this study indicated that the Facing Your Fears-Adolescent Version may be a feasible and acceptable group cognitive behavior treatment program for teens with high-functioning autism spectrum disorders. For this psychiatrically complex group of teens, significant reductions in anxiety symptoms, severity, and interference occurred following the delivery of the FYF-A treatment. However, small sample size and lack of a control group limit the generalization of findings. Future studies should include a more rigorous experimental design (e.g., random assignment, control group, and independent evaluators) to further explore the effectiveness of FYF-A. 

## Figures and Tables

**Table 1:** Participant characteristics (*n* = 24).

Participant characteristic	*M* (range, SD)/Percent
Age	15.5 (13.4–18.0)
Full-scale IQ	100.5 (66–128, SD = 17.27)
Nonverbal IQ	100.2 (65–137, SD = 17.55)
Verbal IQ	103.6 (73–131, SD = 17.23)
Gender	
Male	62.5%
Ethnicity	
Caucasian	66.7%
Other	33.3%
Autism spectrum DX	
Autism	29.2%
Asperger's disorder	54.2%
PDD-NOS	16.7%
Taking Medications	58.3%
Mother's highest level of education	
High school (partial)	4.2%
High school (graduate)	8.3%
College (partial)	25%
College (graduate)	45.8%
Postcollege education	16.6%

**Table 2:** Parent (*n* = 24) and Teen (*n* = 23) Average Satisfaction Ratings of Group Activities: FYF-A.

	Parents	Teens
Treatment overview	4.30 (3–5; SD = .70)	Parents only
What makes me worried worksheet	4.21 (3–5; SD = .72)	3.26 (1–5; SD = 1.32)
How people react when they feel worried	4.13 (1–5; SD = 1.03)	2.96 (1–5; SD = 1.26)
Real danger/false alarm	4.13 (3–5; SD = .80)	3.30 (1–5; SD = 1.22)
Relaxation activity	3.75 (2–5; SD = .99)	3.70 (1–5; SD = 1.26)
Thermometers/PDAs for anxiety ratings	4.17 (2–5; SD = 1.05)	3.48 (1–5; SD = 1.24)
Active minds and helpful thoughts activity	3.90 (1–5;SD = .99)	3.09 (1–5; SD = 1.12)
Finding our target	4.33 (2–5; SD = .82)	3.35 (1–5; SD = 1.23)
Steps to success worksheet	4.46 (3–5; SD = .66)	3.65 (1–5; SD = 1.27)
Overview of exposure	4.38 (3–5; SD = .77)	Parents only
Where do we begin	4.46 (3–5; SD = .59)	3.64 (1–5; SD = 1.22)
Creating graded exposure hierarchies	4.21 (3–5; SD = .72)	3.35 (1–5; SD = 1.19)
Coaching and dyadic work on graded exposure	4.38 (2–5: SD = .82)	3.21 (1–5; SD = 1.28)
Practicing facing fears	4.75 (4–5; SD = .44)	3.35 (1–5; SD = 1.37)
Learning skills video	Teens only	2.52 (1–5; SD = 1.27)
Psychoeducation model of anxiety	4.08 (3–5; SD = .72)	Parents only
Learning skills—practice talking to new people	Teens only	3.43 (1–5; SD = 1.59)
